# Room-Temperature Hydrogen-Sensing Capabilities of Pt-SnO_2_ and Pt-ZnO Composite Nanoceramics Occur via Two Different Mechanisms

**DOI:** 10.3390/nano11020504

**Published:** 2021-02-17

**Authors:** Ming Liu, Pengcheng Li, Yong Huang, Liang Cheng, Yongming Hu, Zilong Tang, Wanping Chen

**Affiliations:** 1 Research Institute of Wuhan University in Shenzhen, Shenzhen 518057, China; 2019282020095@whu.edu.cn (M.L.); 2016282020104@whu.edu.cn (P.L.); hy819456531@126.com (Y.H.); 2 Hubei Key Laboratory of Ferro and Piezoelectric Materials and Devices, Faculty of Physics and Electronic Science, Hubei University, Wuhan 430062, China; 13554149979@163.com (L.C.); huym@hubu.edu.cn (Y.H.); 3 School of Materials Science and Engineering, State Key Laboratory of New Ceramics and Fine Processing, Tsinghua University, Beijing 100084, China; tzl@tsinghua.edu.cn; 4 Key Laboratory of Artificial Micro- and Nano-Structures of Ministry of Education, School of Physics and Technology, Wuhan University, Wuhan 430072, China

**Keywords:** Pt-SnO_2_, Pt-ZnO, composite nanoceramics, hydrogen, mechanism

## Abstract

Impressive room-temperature gas-sensing capabilities have been reported for nanomaterials of many metal oxides, including SnO_2_, ZnO, TiO_2_, WO_3_, and Fe_2_O_3_, while little attention has been paid to the intrinsic difference among them. Pt-SnO_2_ and Pt-ZnO composite nanoceramics have been prepared through convenient pressing and sintering. The former shows strong and stable responses to hydrogen in 20% O_2_-N_2_ (synthetic air) at room temperature, while the responses to hydrogen in N_2_ cannot be stabilized in limited times; the latter shows strong and stable responses to hydrogen in N_2_, while the responses to hydrogen in synthetic air are greatly depressed. Further analyses reveal that for Pt-ZnO, the responses result from the reaction between hydrogen and oxygen chemisorbed on ZnO; while for Pt-SnO_2_, the responses result from two reactions of hydrogen, one is that with oxygen chemisorbed on SnO_2_ and the other is hydrogen chemisorption on SnO_2_. These results reveal two different room-temperature hydrogen-sensing mechanisms among MOXs, which results in highly contrasting room-temperature hydrogen-sensing capabilities attractive for sensing hydrogen in oxygen-contained and oxygen-free environments, separately.

## 1. Introduction

Because of their high sensitivity, simple preparation, good stability, low production cost, and controllable morphology, gas sensors based on SnO_2_ thick films have been successfully commercialized for several decades. However, they all have to work at elevated temperatures (~500 °C) [1], which leads to increased energy consumption, shortened service life, and increased safety risks [2]. In order to develop room-temperature metal oxide (MOX) gas sensors, nano-materials of various MOXs have been synthesized and impressive room-temperature gas-sensing capabilities have been observed for many of them, including SnO_2_ [3], TiO_2_ [4,5], WO_3_ [6], Fe_2_O_3_ [7,8], ZnO [9,10,11,12,13,14]. It is generally believed that the large specific surface of nano-structured MOXs is the key for them to be room-temperature gas sensitive. However, highly impressive room-temperature hydrogen-sensing capabilities have been observed in Pt-WO_3_ and Pt-Nb_2_O_5_ composite ceramics with 1.5–2.5 µm WO_3_ grains and 1–2.5 µm Nb_2_O_5_ grains [15,16], respectively, and the catalytic role of Pt has been proven responsible for both kinds of composite ceramics to respond strongly to hydrogen at room temperature. As for the advantages of nano-structured MOXs, their large number of point defects, especially oxygen vacancies, have been found to play a vital role in enhancing their room-temperature gas sensitivities [17,18].

For sensing reducing gases in air by n-type MOXs, a well-known mechanism is established as follows [19,20]: oxygen is chemisorbed on MOXs, which captures electrons from MOXs and an electron-deficient layer, or depletion layer is formed beneath the surface of MOXs. When a reducing gas reacts with chemisorbed oxygen, the electrons are returned to MOXs and the resistance is decreased. This mechanism was first established to account for gas sensing by MOXs at elevated temperatures, and in recent years, it has been widely adopted to account for room-temperature gas-sensing capabilities revealed in various nanostructured MOXs, and much attention has been paid to decrease the grains of MOXs to sizes comparable to the thickness of depletion layer to achieve high sensitivities [21,22,23]. It should be pointed out, however, that the reaction between reducing gases and MOXs may strongly depend on the temperature and room-temperature gas-sensing mechanism may be different from that at elevated temperatures for MOXs. Up to date, the reaction between reducing gases and MOXs at room temperature has been actually quite neglected, and the difference between different MOXs has rarely been investigated, either. As a matter of fact, highly contrasting room-temperature hydrogen-sensing capabilities have already been observed for Pt-TiO_2_ and Pt-SnO_2_ composite nanoceramics prepared in the same way [24], which suggests that the room-temperature hydrogen-sensing mechanism should be studied for various MOXs individually.

As two typical MOXs for gas sensing, SnO_2_ and ZnO have been chosen for their room-temperature hydrogen-sensing mechanism to be studied in-depth in this study. Pt-SnO_2_ and Pt-ZnO composite nanoceramics were first prepared through convenient pressing and sintering, and their room-temperature hydrogen-sensing characteristics were then investigated. As bulk materials prepared through pressing and sintering, they have such advantages of low fabrication cost, high mechanical robustness, and high thermal stability over those one and two-dimensional nanostructured MOXs. Roughly speaking, Pt-SnO_2_ was found attractive for sensing hydrogen in oxygen-contained atmospheres while Pt-ZnO was capable for sensing hydrogen in oxygen-free atmospheres. Two different room-temperature hydrogen-sensing mechanisms were further revealed for them: for Pt-ZnO, hydrogen only reacts with chemisorbed oxygen on ZnO at room temperature; while for Pt-SnO_2_, hydrogen not only reacts with chemisorbed oxygen on SnO_2_ but also is chemisorbed on SnO_2_ at room temperature by itself. Obviously, these different mechanisms are responsible for the highly contrasting room-temperature hydrogen-sensing characteristics observed for them.

## 2. Materials and Methods

SnO_2_ nanoparticles (70 nm), ZnO nanoparticles (50 nm), and Pt particles (~1 μm), all from Aladdin, Shanghai, China, were used as the starting materials. First of all, SnO_2_ nanoparticles and Pt particles were dispersed into deionized water at a weight ratio of 99:1. Then the mixtures were stirred for 4 h on a magnetic stirrer, next dried in an oven at 120 °C for 12 h. After that a pressure of about 4 MPa was applied by a hydraulic press to press the dry powders into pellets with a diameter of about 10 mm and a thickness of about 1 mm. Pellets of Pt-ZnO were prepared in the same way starting from ZnO nanoparticles and Pt particles. A series of sintering temperatures were investigated for both kinds of nanoceramics, and the pellets of Pt-SnO_2_ sintered at 825 °C in air for 2 h showed the best performance among the Pt-SnO_2_ nanoceramics, and the Pt-ZnO pellets sintered at 700 °C showed the best performance among the Pt-ZnO nanoceramics, which were systematically investigated in this study. A pair of rectangular Au electrodes was coated on the main surface of the pellets by DC magnetron sputtering to facilitate gas-sensitive measurement.

A commercial gas sensor measurement system (GRMS-215, Partulab Com., Wuhan, China) [4] was used to measure the hydrogen-sensing characteristics of the sintered samples. During the measurement, the room temperature was maintained at 25 °C, and the relative humidity (RH) in air was maintained at about 50%.

An X-ray diffractometer (BRUKER AXS D8 ADVANCE) was used to perform phase identifications using Cu K_α_ radiation. A scanning electron microscopy (SIRION TMP) was used to conduct microstructural observations. Composition analyses were obtained through energy dispersive spectroscopy (EDS) using OXFORD Aztec 250 instrument.

## 3. Results and Discussions

**Figure 1:**
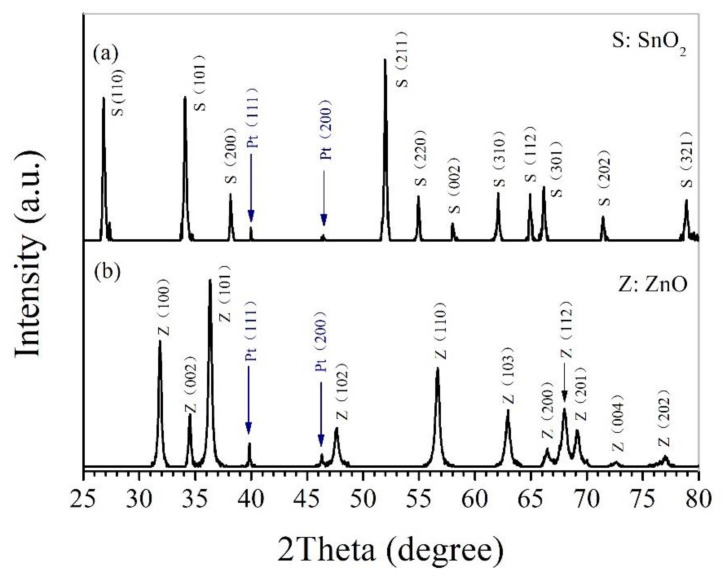
X-ray diffraction patterns taken for the surface of (**a**) Pt-SnO_2_ composite nanoceramics with 1 wt% Pt sintered at 825 °C and (**b**) Pt-ZnO composite nanoceramics with 1 wt% Pt sintered at 700 °C.

Figure 1 shows the X-ray diffraction patterns taken for the surface of Pt-SnO_2_ and Pt-ZnO nanoceramics with 1 wt% Pt sintered at 825 and 700 °C, respectively. As marked in Figure 1a, it is obvious that there are two peaks from metallic Pt, all other peaks belong to SnO_2_, which exists in a rutile phase according to JCPDS file No. 41-1445. Two peaks from metallic Pt can also be observed in Figure 1b, and all other peaks are from ZnO, which exists in hexagonal phase according to JCPDS file No. 36-1451. Hence what we prepared in this study are composites of SnO_2_ and Pt, and ZnO and Pt, separately. It is reasonable that Pt exists in metallic state in those nanoceramics due to the high stability of Pt [4,15,24].

**Figure 2:**
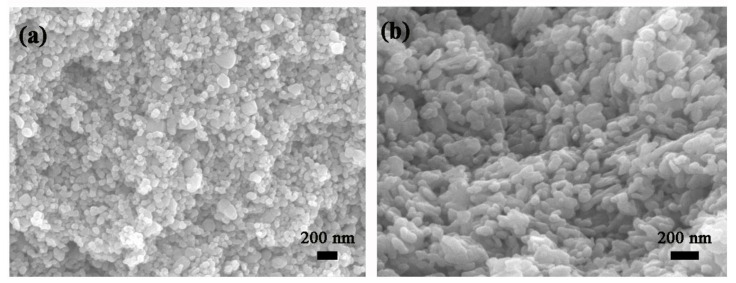
(**a**) SEM micrography of Pt-SnO_2_ composite nanoceramic sintered in the air at 825 °C for 2 h. (**b**) SEM micrography of Pt-ZnO composite nanoceramic sintered in the air at 700 °C for 2 h.

Figure 2 shows SEM micrographs of Pt-SnO_2_ and Pt-ZnO composite nanoceramics, separately. For Figure 2a, it can be seen that most grains are around 70 nm in size, which are the same as that of the as-received SnO_2_ nanoparticles, indicating no obvious grain growth in the sintering. As shown in Figure 2b, ZnO grains are quite non-uniform, some are around 50 nm while many other grains are much larger, indicating that some grain growth has occurred in local areas during the sintering. For both kinds of composite nanoceramics, there is no noticeable shrinkage in diameter after sintering. As a matter of fact, densification should be avoided for the sintering of ceramics intended for gas-sensing applications [5].

**Figure 3:**
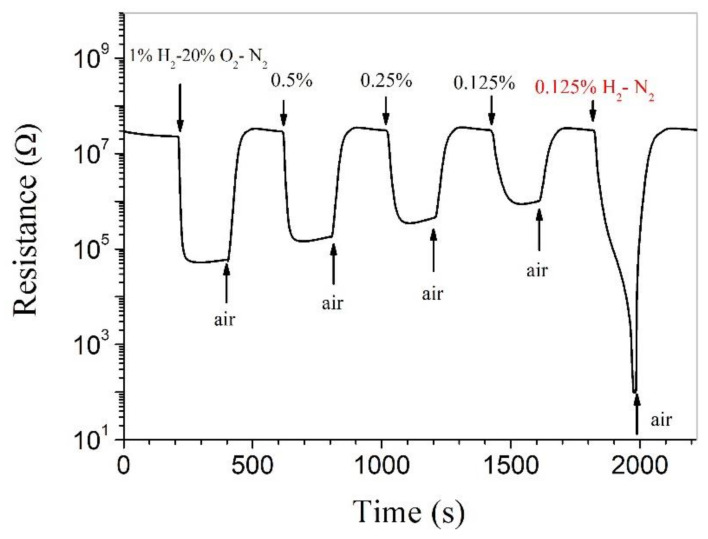
Room-temperature responses to 1%, 0.5%, 0.25%, 0.125% H_2_ in synthetic air (20% O_2_-N_2_) and to 0.125% H_2_ in N_2_, separately, for Pt-SnO_2_ composite nanoceramic sintered at 825 °C.

Highly attractive room-temperature hydrogen-sensing capabilities have been reported for Pt-SnO_2_ composite nanoceramics [24]. As shown in Figure 3, such an attractive room-temperature hydrogen-sensing capability has also been obtained for the Pt-SnO_2_ composite nanoceramics prepared in this study. For H_2_ over the range from 1% to 0.125% in 20% O_2_-N_2_ (synthetic air), the resistance of the sample decreases quickly upon being exposed to hydrogen and becomes stable in limited time. When the concentration of H_2_ decreases from 1% to 0.125%, the resistance decrease, or the response decreases monotonously. As the content of O_2_ in air is around 20%, these results suggest that Pt-SnO_2_ composite nanoceramics are able to sense hydrogen of a range of concentrations in air at room temperature. On the other hand, for 0.125% H_2_ in N_2_, the resistance decreases sharply with increasing time until goes beyond the measuring limit, as shown in Figure 3. Obviously, this response forms a sharp contrast with that for 0.125% H_2_ in synthetic air and Pt-SnO_2_ composite nanoceramics therefore should not be able to sense hydrogen in oxygen-free environments at room temperature.

**Figure 4:**
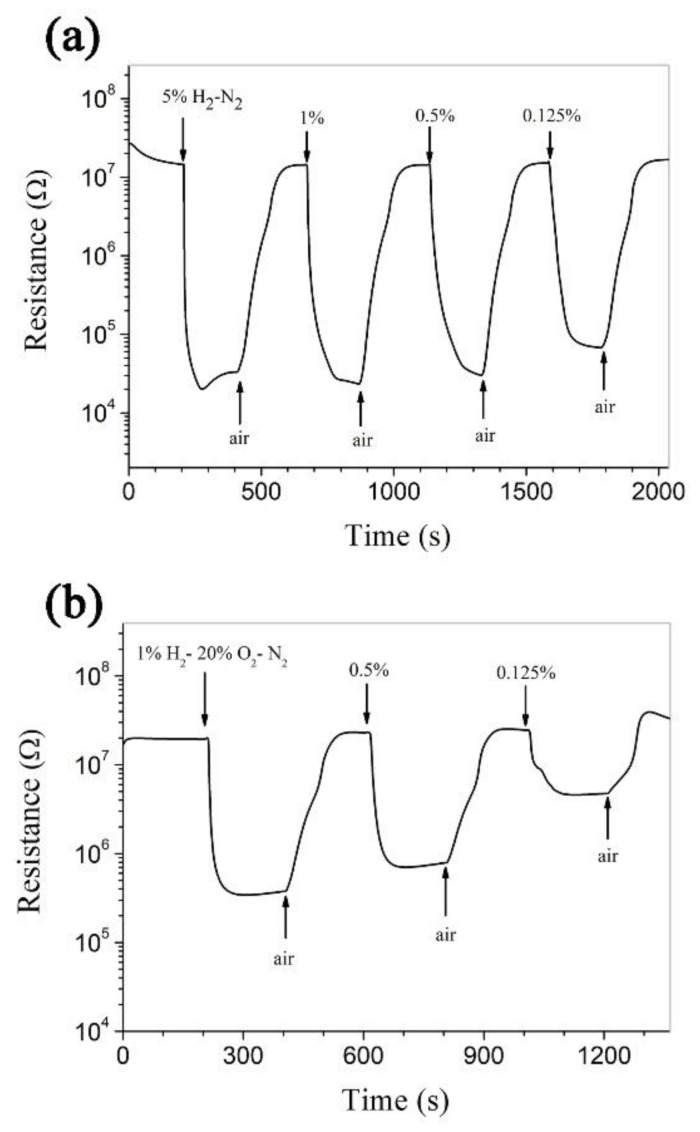
Room-temperature responses (**a**) to 5%, 1%, 0.5%, and 0.125% H_2_ in N_2_, and (**b**) to 1%, 0.5%, and 0.125% H_2_ in synthetic air (20% O_2_-N_2_), separately, for Pt-ZnO composite nanoceramic sintered at 700 °C.

The room-temperature responses of Pt-ZnO composite nanoceramics to hydrogen in N_2_ and hydrogen in synthetic air are shown in Figure 4. As shown in Figure 4a, the resistance of the Pt-ZnO composite nanoceramic sample decreases rapidly with time upon being exposed to hydrogen in N_2_ and becomes stable in a limited time. The sensitivity of a gas sensitive material is usually defined as R_a_/R_g_, where R_a_ and R_g_ are the electrical resistances of the material in clean air and in the measuring gas, respectively [4]. It can be calculated that the sensitivity of the sample is 733, 623, 483, and 208 to 5%, 1%, 0.5%, and 0.125% H_2_ in N_2_, respectively. It is clear that Pt-ZnO composite nanoceramics are able to sense hydrogen in oxygen-free atmospheres at room temperature, where hydrogen sensors based on Pt-SnO_2_ composite nanoceramics cannot be applied. As shown in Figure 4b, the sensitivity of the sample is 60, 35, and 5 to 1%, 0.5%, and 0.125% H_2_ in synthetic air, respectively. Compared with Pt-SnO_2_ composite nanoceramics, Pt-ZnO composite nanoceramics show a much smaller sensitivity to the same concentration of hydrogen in synthetic air. In short, Pt-ZnO composite nanoceramics are attractive for sensing hydrogen in oxygen-free atmospheres, while Pt-SnO_2_ composite nanoceramics are attractive for sensing hydrogen in air.

**Figure 5:**
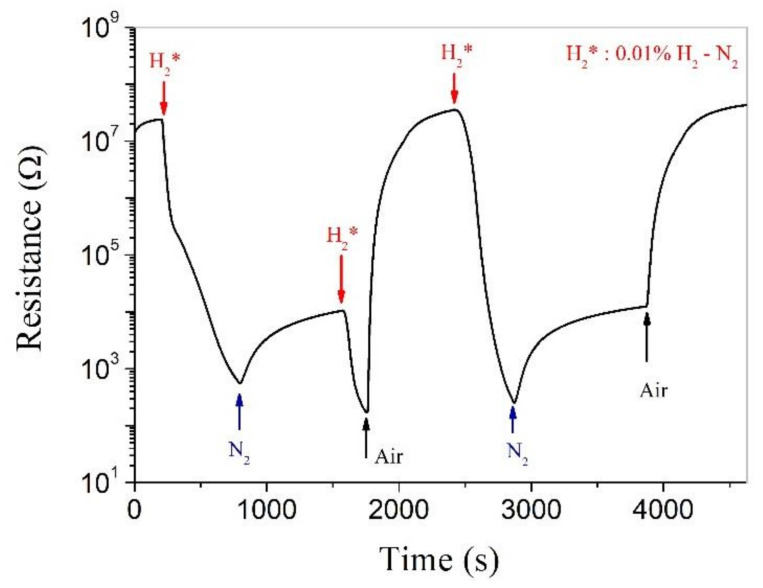
Real-time electrical resistance response of a Pt-SnO_2_ composite nanoceramic pellet, sintered at 825 °C and coated with a pair of Au electrodes, to different kinds of atmospheres at room temperature.

To study the hydrogen-sensing mechanisms for Pt-SnO_2_ and Pt-ZnO composite nanoceramics, we have measured their responses to some special atmospheres in certain well-designed sequences. For Pt-SnO_2_ composite nanoceramics, it has been concluded that hydrogen can be chemisorbed on SnO_2_ at room temperature in the presence of Pt [24]. Figure 5 shows the measuring result obtained for a Pt-SnO_2_ composite nanoceramic sample. The resistance of the sample decreases sharply with increasing time in flowing 0.01% H_2_-N_2_. When the atmosphere is changed to N_2_, a turning point appears and the resistance increases gradually with time in flowing N_2_. Both the decrease and the increase in resistance can be well explained in terms of hydrogen chemisorption on SnO_2_: in flowing 0.01% H_2_-N_2_, more and more hydrogen atoms are chemisorbed on SnO_2_ and so the resistance of SnO_2_ decreases continuously; when the atmosphere is changed to N_2_, hydrogen atoms chemisorbed on SnO_2_ desorb as hydrogen molecules and the electrons they have donated to SnO_2_ leave with them, so the resistance increases with time in flowing N_2_.

As shown in the second cycle, when the sample is kept in flowing N_2_ for a relatively long time and the resistance of the sample is almost stabilized, indicating that the hydrogen desorption has gradually finished. However, the resistance at this state is still much smaller than the initial resistance in air, and when the atmosphere is then changed from N_2_ to air, the resistance is increased quickly until its initial value in air. These results suggest that in flowing 0.01% H_2_-N_2_, hydrogen must have two kinds of reactions with SnO_2_: one is the reaction with SnO_2_, or hydrogen chemisorption on SnO_2_, and the other is the reaction with oxygen chemisorbed on SnO_2_. When the atmosphere is changed from 0.01% H_2_-N_2_ to N_2_, oxygen chemisorption destroyed by hydrogen cannot be restored and so the resistance cannot be fully recovered; while for the recovery in air, oxygen not only reacts quickly with hydrogen chemisorbed on SnO_2_ but also is chemisorbed on SnO_2_ by itself, so the resistance is fully recovered to its initial state in air.

The room-temperature hydrogen-sensing capability of SnO_2_ is thus related to both oxygen adsorption and hydrogen adsorption. Under anaerobic conditions, more and more hydrogen atoms are chemisorbed on SnO_2_, and the resistance of SnO_2_ becomes smaller and smaller, which cannot be stabilized in a limited time. In the case of hydrogen in air, the surface of SnO_2_ has both hydrogen adsorption and oxygen adsorption. The former contributes electrons to the conduction band of SnO_2_, and the latter captures electrons from SnO_2_, which finally reach equilibrium and the resistance of SnO_2_ can be stabilized in a limited time. Therefore, the Pt-SnO_2_ system can effectively detect hydrogen in an aerobic environment, but it is unable to detect hydrogen in an oxygen-free environment.

**Figure 6:**
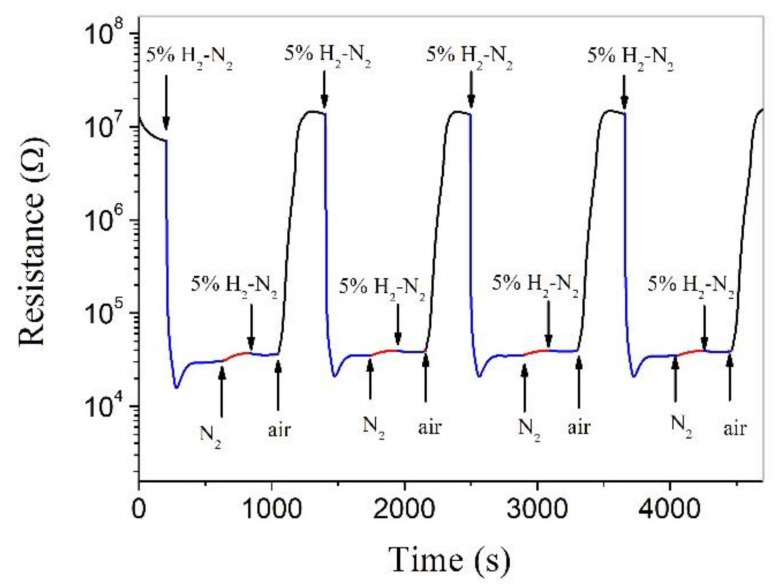
Real-time electrical resistance response of a Pt-ZnO composite nanoceramic pellet, sintered at 700 °C and coated with a pair of Au electrodes, to different kinds of atmospheres at room temperature.

Figure 6 shows the responses to some special atmospheres in a certain sequence for a Pt-ZnO composite nanoceramic sample, which forms a sharp contrast with Figure 5 mainly in two respects. First, the resistance of the sample can be stabilized in flowing 5% H_2_-N_2_ in a limited time, while in Figure 5, the resistance of the sample cannot be stabilized in a very low concentration of hydrogen (0.01% H_2_-N_2_). Second, the sample shows no responses in its resistance to subsequent changes in the surrounding atmosphere, from 5% H_2_-N_2_ to N_2_, and then from N_2_ to 5% H_2_-N_2_. From these results, we can infer that the hydrogen-sensing mechanism of ZnO is as follows: in flowing 5% H_2_-N_2_, hydrogen reacts with adsorbed oxygen on the surface of ZnO, which release the electrons captured by oxygen to ZnO and the resistance of ZnO is decreased. After the reaction is finished for this concentration of hydrogen, the resistance is stabilized. There must be no hydrogen chemisorption on ZnO, and the resistance of the sample thus remains unchanged when its surrounding atmosphere is changed from 5% H_2_-N_2_ to N_2_, and then from N_2_ to 5% H_2_-N_2_. Finally, when air is introduced, oxygen is chemisorbed on ZnO and the resistance is fully recovered. In this way, Pt-ZnO system is able to detect hydrogen in an oxygen-free environment.

**Figure 7:**
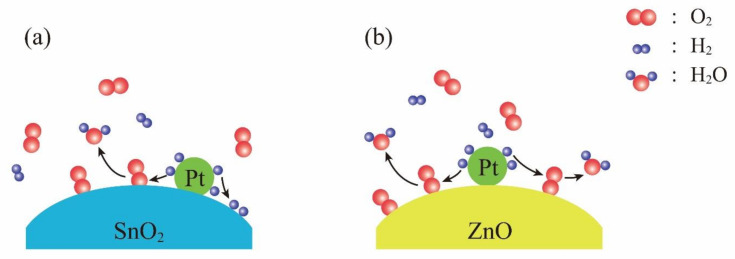
Schematic illustrations for room-temperature hydrogen-sensing mechanisms of (**a**) Pt-SnO_2_ composite nanoceramics, and (**b**) Pt-ZnO composite nanoceramics.

It is interesting to compare the room-temperature hydrogen-sensing mechanisms between Pt-SnO_2_ and Pt-ZnO composite nanoceramics. As shown in Figure 7a, for Pt-SnO_2_ composite nanoceramics, hydrogen molecules are split into atoms by Pt, which are highly reactive and some of them react with oxygen chemisorbed on SnO_2_, and the others are chemisorbed on SnO_2_. Both kinds of reactions release electrons to SnO_2_ and for hydrogen in N_2_, due to hydrogen chemisorption, the resistance of SnO_2_ cannot be stabilized even when all chemisorbed oxygen has been reacted by hydrogen. For Pt-ZnO composite nanoceramics, on the contrary, there is no hydrogen chemisorption on ZnO, as shown in Figure 7b. The resistance of ZnO will be stabilized when the reaction between chemisorbed oxygen and hydrogen atoms is finished, in this way they show stable responses to hydrogen in N_2_. Obviously, some intrinsic differences between SnO_2_ and ZnO are responsible for their different reactions with hydrogen atoms, which result in highly contrasting room-temperature hydrogen-sensing characteristics between Pt-SnO_2_ and Pt-ZnO composite nanoceramics.

## 4. Conclusions

Pt-SnO_2_ and Pt-ZnO composite nanoceramics have been prepared through convenient pressing and sintering. These two typical MOXs exhibit different behaviors to hydrogen in different atmospheres. For Pt-SnO_2_, its response to hydrogen in synthetic air is strong and stable, but its response to hydrogen in N_2_ cannot be stabilized. For Pt-ZnO, it shows strong and stable responses to hydrogen in N_2_. Further study shows that the room-temperature hydrogen-sensing capability of Pt-SnO_2_ is determined by both oxygen chemisorption and hydrogen chemisorption while that of Pt-ZnO is determined by oxygen chemisorption alone. These results clearly show that the room-temperature gas-sensing mechanism of one MOX can be basically different that of another, which leads to contrasting room-temperature gas-sensing capabilities among MOXs.

## Data Availability

The data presented in this study are available on request from the corresponding author.
